# Quantitative analysis of mental and spiritual policy texts relevant for aging well in China

**DOI:** 10.3389/fpubh.2025.1522971

**Published:** 2025-04-08

**Authors:** Liyan Zhang, Mingyang Qin

**Affiliations:** School of Law and Politics, Liaoning Normal University, Dalian, China

**Keywords:** spiritual older adult care, policy texts, policy tools, policy feedback, aging, mental health

## Abstract

The policies related to spiritual older adult care issued by the state since the 18th National Congress were selected as the research object, and a two-dimensional analysis framework composed of policy tools and policy objectives was established. Seventy-one policy documents were analyzed by the content analysis method, and 285 analysis units were frequency counted to analyze the value tendency and inherent deficiencies of the current spiritual older adult care policies in depth. The policy tool dimension is characterized by the uncoordinated internal structure of supply-type tools, the varying level of environment-type tools, and the relative shortage of demand-type tools, and there is a gap in the degree of policy emphasis in the policy objective dimension. In the light of the policy feedback theory, we explore the policy recommendations that are conducive to promoting the development of mental older adult care from the perspective of the resource effect, the explanatory effect, the evolutionary effect, and the learning effect in turn.

## 1 Introduction

Rapid socio-economic and technological development has contributed to the increasing longevity of human beings, but at the same time the fertility rate has also declined, and as early as in the 19th century, France and Sweden were the first to embark on the journey of population aging. Since the 1970s, population aging has become a widespread global problem. According to the United Nations, the proportion of people aged 65 and above will be about 10.5% globally in 2024, and it is expected to climb to 16% in 2050, with the population aged 65 and above accounting for one-third of the total global population. The proportion of older adult people is increasing in countries around the world, and China is also facing the trend of aging. During the “14th Five-Year Plan” period, the degree of population aging in China will be transitioned from mild to moderate. According to the latest forecast data released by the National Bureau of Statistics, by the time the objectives of the “14th Five-Year Plan” are generally completed, the size of the older adult population aged 65 years old and above will be as high as 210 million, and by 2050, the number of older adult people aged 60 years old and above in China will exceed 500 million. Along with the realization of the goal of building a moderately affluent society in all aspects, the demand structure of the older adult population has gradually shifted from the material security type that supports life to the spiritual and cultural type that seeks development. More and more older people desire spiritual support, and the development of a spiritual older adult care support system to better meet the spiritual needs of the older adult population is a social issue that needs to be addressed urgently (1). Common psychological problems in the older adult include loneliness, anxiety, depression, sleep disorders, dementia, cognitive impairment, worry and suspicion, stubbornness and conservatism, obedience and dependence, etc. (2). Golden et al. (3) found that older people are in urgent need of psychosocial support, and that loneliness and social networks have an independent impact on the mood and well-being of older people living in the community through a study of 1,299 older people. Currently, 33.1% of Chinese older adults over the age of 60 suffer from depression, and as a marginalized group in society whose loneliness and depression are not known, failure to effectively respond to the spiritual needs of older adults will certainly increase the burden on society and thousands of families (4). According to the Notice on Carrying Out older adult Mental Care Actions, the National Health Commission plans to carry out large-scale older adult mental care actions throughout the country from 2022 to 2025, with the aim of gaining a deeper understanding of the mental health status of the older adult and their needs, increasing the concern of the older adult for mental health, and improving the professional capacity of grass-roots service personnel in mental health.

Spiritual well-being as an inner strength is one of the important dimensions of human health, and in Western countries spiritual well-being has been categorized into two types: religious well-being landscapes, which is the human understanding of the health of the spiritual life in connection with supernatural forces (5); and existential well-being landscapes, which refers to the psychological anxiety of being in the midst of one’s social life. The human spirit is considered to be the essence of existence, inspiring and guiding us to live a meaningful life, and as holistic health becomes popular in geriatric nursing, the balance and connection between body, mind, and spirit becomes especially important for older individuals (6), but there is little evidence in the relevant nursing literature on how nurses of older adults respond to their spiritual needs (7). Foreign scholars have focused on the quality of life or spiritual care of the older adult from a spiritual or religious-spiritual perspective (8), while domestic scholars have focused on research from a social life and psychological perspective (9), based on which spiritual older adult care is defined as the provision of support and care for the older adult by the family, the society, and the government at the spiritual level, so that the spiritual needs of the older adult individuals can be met as well as their material needs, and the specific implementation process includes multi-dimensional social participation mechanisms such as cultural activities, social interactions, emotional exchanges and self-realization, and the construction of an age-friendly social environment in the social atmosphere, so as to enhance the sense of well-being and sense of achievement of the older adult (10).

Spiritual care helps older adults develop spirituality and slow aging (11), and promotes mental health and well-being (12). Spiritual care increases older adults’ trust in the healthcare team, which increases their satisfaction and is vital to people’s lives (13). Ai (14) emphasizes the importance of spiritual well-being and spiritual growth to the care and research of the older adult population by developing a discussion of the relationship between disadvantage, spiritual growth, and spiritual well-being from an interfaith and interdisciplinary perspective. Specialized care for the spiritual needs of older adults is advocated when they face negative life circumstances. In a study conducted by the Mental Health Association, 87% of respondents reported that spiritual care met their needs and was a positive experience (15). Research on China’s aging policy from the perspective of the spiritual needs of the older adult has many advantages. First, it can plan ahead, rationalize the layout, formulate policies that are compatible with demographic changes, ease the contradiction between the supply and demand of social resources, and ensure the smooth operation of society. Secondly, it is committed to providing comprehensive protection for the older adult, meeting their medical needs, enriching their spiritual and cultural life, and improving their quality of life and sense of well-being in all aspects, so that they can have a full and happy old age. Thirdly, it is to enable the older adult to share the fruits of social development, promote intergenerational understanding and support, create a harmonious social atmosphere, and safeguard the long-term stability of society.

Policy feedback theory, as a new way of policy analysis (16), focuses on highlighting the structuring role of policy and its shaping of target group attitudes and behaviors (17), which argues that the formulation and implementation of public policy can both give resources and explanations to citizens and shape their attitudes and behaviors (18), and believes that the past policies will implicitly influence the formulation and implementation of future policies, and the core idea is that old policies influence and shape future politics and policies (19), to which Pearson proposes four kinds of policy feedback effects: the resource effect, the explanatory effect, the evolutionary effect and the learning effect.

Against the macro backdrop of the increasing trend of global population aging, research on aging policies has become an important issue common to all countries in the world. Although the aging process varies from country to country and region to region, they are all facing the challenge of how to meet the growing and complex needs of the older adult. In this environment, China has actively responded to and implemented a national strategy to cope with population aging. In this context, the design of policies on aging is of paramount importance, and needs to be closely adapted to the new characteristics of the needs of the older adult, and adjusted and optimized to keep pace with the times, so as to better promote the cause of the older adult in the new era toward a new journey of high-quality development, and to echo the global common search for effective responses to the aging society, so as to create a better quality of life environment for the older adult groups. At this stage, the implementation of the older adult policy involves a wide range of fields, but the content of the spiritual older adult care protection system for the older adult is more limited, considering that China’s spiritual older adult care is still in the primary stage, the academic research on related policies is more limited, first, most of the main objects of the research on spiritual older adult care policy are focused on the rural older adult and the empty-nested older adult, and there are fewer researches on the urban and other old age groups, and the policy research lacks wholeness and universality (20). Secondly, although the existing policy research focuses on the construction of the spiritual older adult care support system from the macro level, the research in the micro area is still relatively small, ignoring the accessibility of spiritual older adult care services for the older adult, and the coverage of spiritual older adult care service policies is limited (21). Based on the policy text analysis to provide a new research perspective for the development of spiritual older adult care in China, based on the quantitative study of spiritual older adult care policy text (22), we construct a two-dimensional analysis model of policy tools – policy objectives from the vertical perspective, and carry out the word frequency statistics from the horizontal perspective to identify the high-frequency words in the policy text in order to present the policy text content more comprehensively (23). Finally, the four effects of policy feedback are utilized to propose corresponding countermeasures to the problems embodied in the current policy text, with a view to making future policy formulation and implementation play a positive effect (24).

## 2 Research process

### 2.1 Sources and selection of policy texts

The policies selected for this study are the policy texts on spiritual older adult care at the national level since the 18th National Congress of the Communist Party of China (CPC), and a variety of search methods are used to maximize access to the relevant policies (25), mainly by checking the official websites of the State Council, the Ministry of Civil Affairs, the National Working Committee on the older adult, and the National Health and Health Commission, At the same time, with the help of policy and regulation databases such as Beida Faber, we searched the database by keywords such as “spiritual older adult care,” “psychology of the older adult,” “spiritual comfort,” “hospice,” and so on. Selected policy texts were screened article by article through reading and analysis, and policy contents with little relevance to spiritual older adult care were eliminated. As of December 2024, a total of 71 policies and regulations were screened and included (Table 1).

**Table 1:** List of textual elements of our spiritual older adult care policy, 2013–2024 (filtered).

Number	Issuance date	Policy name	Issuing authority
1	Nov. 14, 2012	The Guiding Opinions of the Ministry of Civil Affairs and the Ministry of Finance on Government Purchase of Social Work Services	Ministry of Civil Affairs
2	Dec. 1, 2012	The Notice of the State Council on Issuing the ‘12th Five-Year Plan for the Development of the Service Industry’	The State Council
3	Dec. 28, 2012	Law of the People’s Republic of China on the Protection of the Rights and Interests of the older adult (2012 Revision)	The Standing Committee of the National People’s Congress
4	July 4, 2013	The Notice of the National Committee on Aging about Conducting the ‘Respect the older adult Month’ Activities in 2013	National Committee on Aging
5	Sep. 6, 2013	Some Opinions of the State Council on Accelerating the Development of older adult Care Service Industry	The State Council
6	Nov. 15, 2013	Opinions of the Ministry of Civil Affairs and the Ministry of Finance on Accelerating the Advancement of Community Social Work Services	Ministry of Civil Affairs
7	Dec. 19, 2013	The Notice of the Ministry of Civil Affairs on Further Strengthening Work Safety and Security Guarantee	Ministry of Civil Affairs
8	Apr. 24, 2015	Law of the People’s Republic of China on the Protection of the Rights and Interests of the older adult (2015 Amendment)	The Standing Committee of the National People’s Congress
9	July 30, 2015	The Notice of the National Committee on Aging about Conducting the 2015 National ‘Respect the older adult Month’ Activities	National Committee on Aging
10	Nov. 18, 2015	The Notice of the General Office of the State Council on Transmitting the Guiding Opinions of the National Health and Family Planning Commission and Other Departments Regarding Promoting the Integration of Medical and Health Care with older adult Care Services	General Office of the State Council
11	Nov. 19, 2015	Guiding Opinions of the General Office of the State Council on Accelerating the Development of Life Service Industry to Promote the Upgrading of Consumption Structure	General Office of the State Council
12	Apr. 1, 2016	Notice of the General Office of the National Health and Family Planning Commission on Printing and Distributing the Division of Key Tasks for the Integration of Medical Care and older adult Care	National Health and Family Planning Commission
13	Oct. 9, 2016	Guiding Opinions of the National Office of Aging, the National Development and Reform Commission, the Ministry of Education, etc. on Promoting the Construction of older adult Livable Environment	National Committee on Aging
……	……	……	……
68	Dec. 31, 2023	Opinions of the Ministry of Civil Affairs, the National Development and Reform Commission, the Ministry of Education, the Ministry of Finance, the Ministry of Human Resources and Social Security, the Ministry of Housing and Urban-Rural Development, the Ministry of Agriculture and Rural Affairs, the Ministry of Commerce, the National Health Commission, the State Administration for Market Regulation, the State Taxation Administration, and the National Working Committee on Aging on Strengthening the Construction of older adult Care Service Talent Teams	National Committee on Aging
69	Jan. 11, 2024	Opinions of the General Office of the State Council on Developing the Silver Economy to Enhance the Well-being of the older adult	General Office of the State Council
70	Jan. 23, 2024	Notice of the Ministry of Civil Affairs and the National Data Administration on Organizing and Carrying Out the Pilot Project of the Comprehensive Platform for Basic older adult Care Services	Ministry of Civil Affairs
71	2024.10.18	Notice of the General Office of the National Health Commission on Enhancing the Medical Service Capability of Geriatrics	National Health Commission of the People's Republic of China

### 2.2 Extraction and analysis of policy theme words

In this study, the ROST.CM6 text analysis software was used to process 71 spiritual older adult care policy texts with general processing and lexical processing successively, and finally, word frequency statistics were performed to eliminate some words with weak relevance to spiritual older adult care, and merge similar keywords such as “senior citizen” and “talent,” and a high-frequency keyword list of spiritual older adult care policy texts is summarized (Table 2). As shown in the chart, the frequency of 16 policy keywords is higher than 60, which means that the policies related to spiritual older adult care focus on the development of medical and mental health services, culture and education, and hospice care, etc., and most of them are targeted at the older adult left behind in rural areas. For the development of spiritual older adult care, the construction of social institutions for the older adult and the improvement of education for the older adult still play an important role. At the same time, the extensive participation of grass-roots voluntary organizations has led to a focus on providing spiritual older adult care services with the community as the organizing unit. In addition, the construction of the professional talent team and the rights and interests protection mechanism required for the development of spiritual older adult care remain to be optimized, and there is a need to improve the construction of relevant disciplines to cultivate talents in the field of spiritual older adult care, to establish a sound legal system for the protection of the rights and interests of the older adult, and to build an age-friendly social environment.

**Table 2:** High-frequency keywords for spiritual older adult care policy texts (filtered).

Number	Keyword	Term frequency	Number	Keyword	Term frequency
1	Senior Citizen	716	16	Countryside	62
2	Service	424	17	Family	59
3	Health	186	18	Rehabilitation	58
4	Society	186	19	Talent	54
5	Older adult Care	152	20	Guarantee	45
6	Institution	130	21	Spiritual Comfort	44
7	Psychology	128	22	Nursing	43
8	Education	117	23	Respect for the Older adult	41
9	Care	109	24	Mechanism	36
10	Organization	105	25	Rights and Interests	35
11	Community	102	26	Grassroots	32
12	Hygiene	89	27	Volunteer	32
13	Hospice Care	87	28	Competence	25
14	Culture	71	29	Sports	21
15	Healthcare	66	30	Law	21

### 2.3 Semantic network analysis of policy keywords

**Figure 1:**
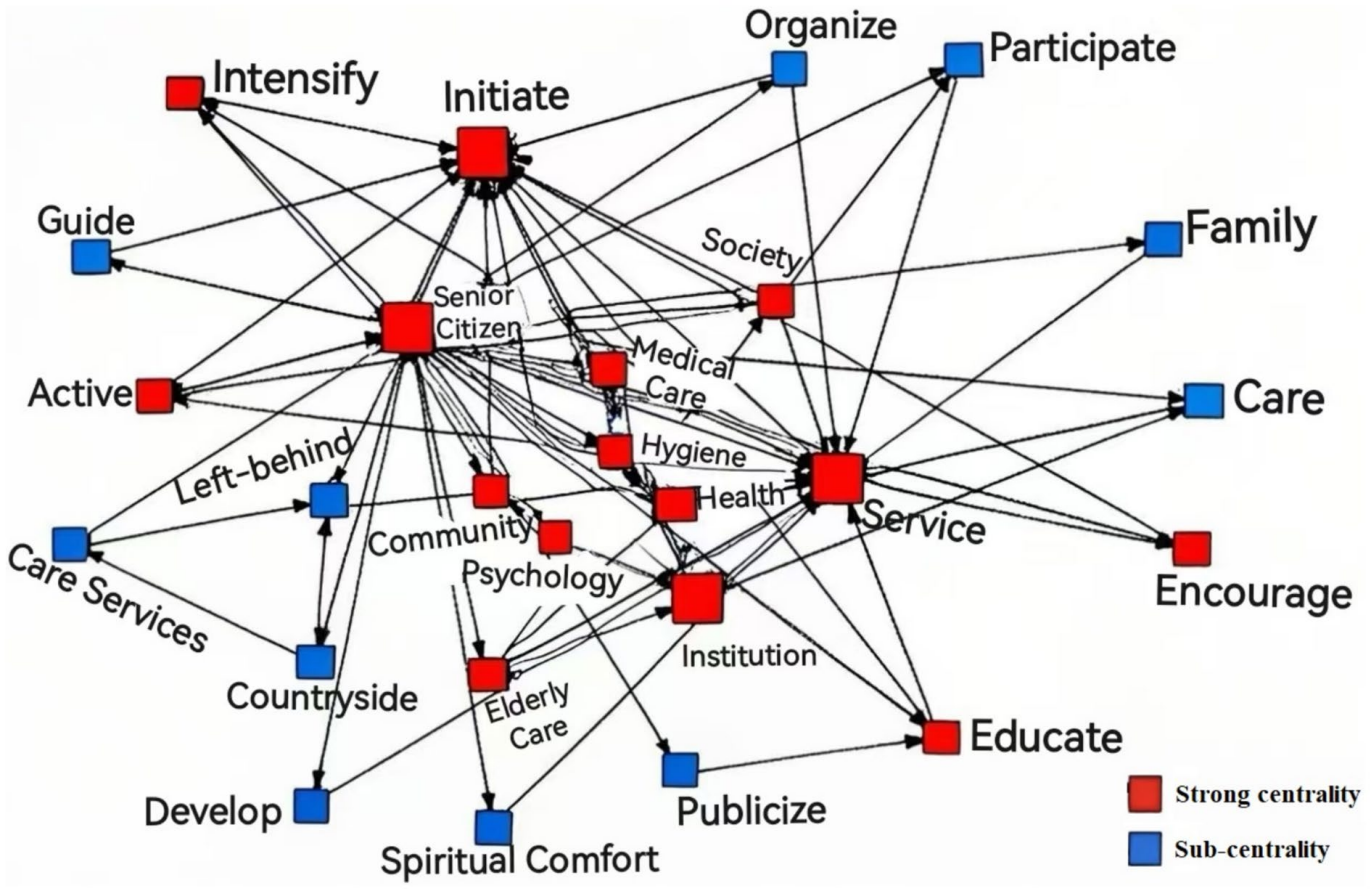
Social semantic network of theme words of China’s spiritual older adult care policy since the 18th National People’s Congress.

Based on the covariance matrix of theme keywords drawn by ROSTCM6 software, a social semantic network diagram of China’s spiritual older adult care policy since the 18th National Congress was drawn with the help of UCINET6 analysis software to present the covariance relationship between the policy keywords, so as to analyze the structural relationship of the textual content of the spiritual older adult care policy (Figure 1). In the social network diagram, each intersection point represents a theme word of the spiritual older adult care policy, and the size of the intersection point can reflect the strength of the centrality of the theme word, which is positively correlated with the importance of the theme word in the text of the policy, and the theme word that is in the center is analyzed, which can help to deeply understand the characteristics and direction of the policy. “Services, institutions, psychology, and health” have strong centrality in the text of the spiritual older adult care policy, reflecting a certain degree of policy content (26). From the figure, it can be seen that since the 18th National Congress, China’s policies related to spiritual older adult care have paid more attention to the integration and development of grassroots social medical and health institutions and mental health services for the older adult, and the establishment of a sound mental health service system and the construction of an age-friendly social environment have become the policy orientation and development trend in the future.

## 3 Analytical framework for spiritual older adult care policy

**Figure 2:**
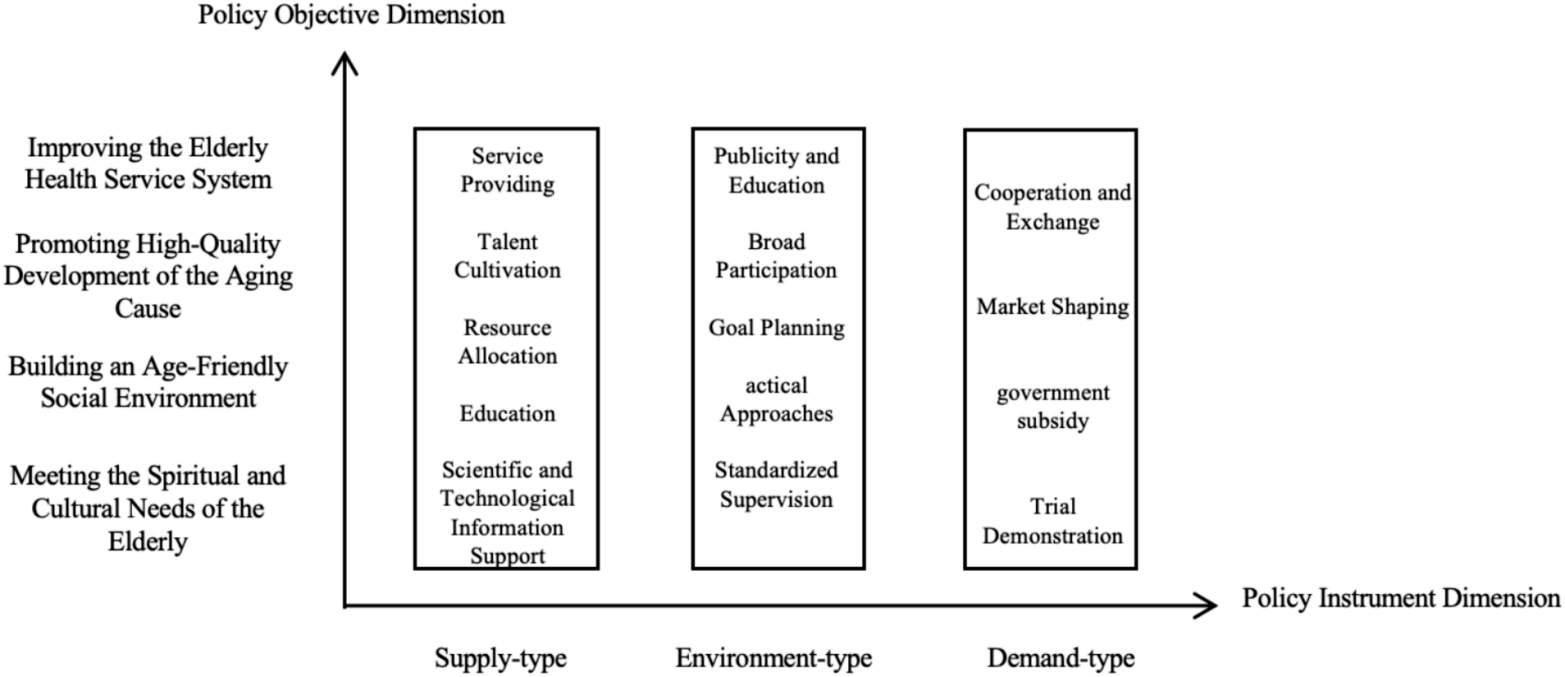
A two-dimensional analytical framework for spiritual older adult care policy.

Policy tools are a kind of means and methods aimed at promoting the realization of policy objectives or solving social problems, and the selection and application of related tools are the key links to ensure the effective implementation of policies. At present, the academic research on policy tools has formed a variety of classification perspectives (27), among which Rothwell and Zegvelad’s classification standard has been most widely used, they classify policy tools into three different categories: supply-type, demand-type and environment-type (28). This classification method not only helps the government’s effective control, but also regulates the market’s value tendency, which is in line with China’s current development trend of constructing a service-oriented government and stimulating market vitality, and is extremely applicable to China’s policy research (29). There are limitations in analyzing the spiritual older adult care policy only by the dimension of a single basic policy tool, which cannot comprehensively reveal the uniqueness and potential deficiencies of the spiritual older adult care policy in various types of policy objectives, and it is also difficult to provide accurate and effective guiding suggestions based on these analyses. Therefore, it is recommended that the four policy objective dimensions be added to construct a two-dimensional analytical framework with the policy tool dimensions in order to analyze the implementation of China’s spiritual older adult care policy from multiple perspectives (Figure 2).

### 3.1 Policy tool dimension (X-dimension)

In the process of promoting the development of spiritual older adult care, three different types of policy tools, namely, supply-type, environment-type and demand-type, all play an important role. Supply-type policy tools are the driving force behind the development of spiritual older adult care, environment-type policy tools provide important support for the steady development of spiritual older adult care, and demand-type policy tools act as “catalysts” for the forward development of spiritual older adult care.

Supply-type policy tools are mainly centered on resource supply. Through the use of sub-tools such as talent cultivation, education and learning, service provision, scientific and technological information support, and resource allocation, the government rationally allocates limited resources and promotes the accelerated flow of various resources to the spiritual older adult care model, so as to provide a strong support for the sustainable development of spiritual older adult care. Environment-type policy tools mainly play an influential role, and the government adopts measures such as publicity and education, strategic measures, standardization and supervision, and target planning to create a good policy environment for the development of spiritual older adult care as well as a social environment with wide participation, so as to escort the construction of an age-friendly social environment. Demand-type policy tools are mainly applied in the market dimension, with the government stabilizing the market environment and broadening the path of development by adopting external means such as cooperation and exchanges, market shaping, government subsidies, pilot demonstrations, and other means, in order to provide pulling power for the high-quality development of the spiritual older adult care business (Table 3).

**Table 3:** Categorization of policy tools and description of their content.

Tool type	Sub-tool content	Tool description	Keyword
Supply-type	Service Providing	Encourage the provision of caring services such as mental health, hospice care, legal aid, or rights and interests protection.	“Care”, “dredge”, “provision”, etc.
	Talent Cultivation	The government implements the talent introduction policy by conducting professional training or offering specialized courses.	“Professional training”, “nurturing talent”, etc.
	Resource Allocation	By offering preferential policies and developing supporting infrastructure, a senior-friendly social environment is established.	“Facilities”, “resources”, “remodel”, “construction”, etc.
	Education	By actively advocating for the provision of educational resources for the older adult, we can promote cultural and healthy aging.	“Health education”, “Geriatric education”, “Culture”, etc.
	Scientific and Technological Information Support	The government provides technological support such as medical care, information, and internet access to support the development of spiritual older adult care	“Science and technology”, “information technology”, etc.
Environment-type	Publicity and Education	Through policy guidance, actively foster a social atmosphere of respecting, caring for, and honoring the older adult throughout society.	“Sensitization”, “education”, “advocacy”, etc.
	Broad Participation	The government encourages the collaborative participation of health institutions, social organizations, enterprises, individuals, and others in providing mental health services for the older adult.	“Voluntary”, “extensive”, “participation”, etc.
	Goal Planning	Conducting an overall planning for the development prospects of spiritual older adult care	“Targeting”, “improving”, “planning”, etc.
	Tactical Approaches	Involving various development-promoting measures such as advancing initiatives, enhancing social participation of the older adult, and standardized construction.	“Support”, “encourage”, “promote”, etc.
	Standardized Supervision	The government ensures sound development through organization building, normative guidance, and supervision and management.	“Regulating”, “managing”, “safeguarding”, etc.
Demand-type	Cooperation and Exchange	Governments at all levels, medical institutions, and older adult care institutions cooperate and exchange ideas on the implementation of mental health services for the older adult.	“Synergistic development”, etc.
	Market Shaping	The government guides and shapes a senior-friendly consumer market environment.	“Consumer sector”, “cultivation”, “new business models” etc.
	Government subsidy	The government purchases and provides mental health-related products and services for the older adult, or alleviates the living pressures of impoverished seniors.	“Financial”, “support”, “input” etc.
	Trial Demonstration	Encourage the establishment of relevant pilot institutions or the conduct of pilot demonstrations to explore models for spiritual older adult care	“Pilot”, “demonstration” etc.

### 3.2 Policy objective dimension (Y-dimension)

Taking into account the development trend of spiritual older adult care in China, the policy objectives for the development of spiritual older adult care in the new era can be categorized into perfecting the health service system for the older adult, promoting the high-quality development of the cause of the older adult, building an age-friendly social environment and meeting the spiritual and cultural needs of the older adult to ensure that they are fully supported in terms of health, economic security and social participation and that the quality of their lives is comprehensively improved.

Improving the health service system for the older adult is to take the actual needs of the older adult as the starting point, accurately position the services, optimize the service supply, and promote the construction of a multi-level health service system for the older adult in order to enhance the effectiveness of social security governance. Promoting the high-quality development of the aging cause is to promote the development of the spiritual older adult care and health service industry through market shaping and industry cultivation, strengthen the training of spiritual older adult care personnel, improve the ability of the older adult to prevent fraud and digital literacy, and better satisfy the needs of the older adult in the level of mental health through the all-around standardization of spiritual older adult care services. The construction of an age-friendly social environment includes building a livable environment for the older adult, optimizing the mechanism for legal aid and protection of the rights and interests of the older adult, promoting intergenerational exchanges and mutual assistance to enhance social harmony, and increasing the assistance provided to the older adult in poverty. Satisfying the spiritual and cultural needs of the older adult mainly includes setting up activity centers for the older adult to carry out cultural and sports activities, opening universities for the older adult to strengthen education for the older adult, and fostering the development of cultural industries in the cultural market, so as to give the older adult a sense of enjoyment and a sense of learning in their old age.

## 4 Text codes for spiritual older adult care policy

Based on the analysis of spiritual older adult care policy tool dimension and policy objective dimension, this study takes the screened 71 policy texts as the basic unit of analysis, and adopts the content analysis method to uniformly code the screened samples according to the form of “policy number-specific articles” (30). In the process of coding, we maximize the coverage of the content of the policy text, and finally form the content analysis unit coding table of the spiritual older adult care policy text (Table 4).

**Table 4:** Table of content codes and excerpts from policy texts.

Number	Policy name	Content analysis unit	Encoding
1	Opinions of the Ministry of Civil Affairs, the Ministry of Public Security, the Ministry of Justice, and Other Departments on Strengthening the Caring Services for Left-behind older adult in Rural Areas	1. Comprehending the Overall Requirements for Caring Services for Left-Behind Older adult in Rural Areas	1–1
		2. Strengthening the primary responsibility of families in the support and care services for left-behind Older adult in rural areas	1–2
		3. Leveraging the role of village committees in safeguarding the rights and interests of left-behind older adult in providing care and support services in rural areas.	1–3
		4. Utilizing the unique role of aging-focused organizations and facilities in providing care and support services for left-behind older adult in rural areas.	1–4
		5. Encouraging widespread participation of social forces in caring for and supporting left-behind older adult individuals.	1–5
		6. Strengthening government support and safeguards for caring and support services aimed at left-behind older adult in rural areas.	1–6
…	……	……	……
69	Opinions of the General Office of the State Council on Developing the Silver Economy to Enhance the Well-being of the older adult	1. Optimizing older adult health services.	69–1
		2. Enriching cultural and sports services for the older adult	69–2
		3. Launch a high-standard navigation action	69–3
		4. Building a New Format of Intelligent & Health Care for the Older adult	69–4
		5. Diversifying the Tourism Service Landscape	69–5
		6. Cultivating Older adult Human Resources, ……	69–6
		7. Crack down on fraud targeting the older adult	69–7
70	Notice of the Ministry of Civil Affairs and the National Data Administration on Organizing and Carrying Out the Pilot Project of the Comprehensive Platform for Basic older adult Care Services	1. Vigorously promote “Internet Plus Regulation”	70–1
71	Notice of the General Office of the National Health Commission on Enhancing the Medical Service Capability of Geriatrics	3. Carry out geriatric diagnosis and treatment services in a standardized manner	71–1

## 5 Content analysis of spiritual older adult care policy based on policy tools

Based on the two-dimensional analytical framework of spiritual older adult care, the 285 valid codes were accurately categorized and scientifically counted with reference to the interpretation of the indicators of the policy tool and goal dimensions, and the frequency distribution table of the use of policy tools was drawn accordingly (Table 5). From the results, it can be seen that there are differences in the degree of preference for the use of basic policy tools, and the focus of policies on the policy objective dimension is also different.

**Table 5:** Frequency distribution of use of policy tools.

Tool type		Improving the older adult health service system	Promoting high-quality development of the aging cause	Building an age-friendly social environment	Meeting the spiritual and cultural needs of the older adult	Total	Proportion (%)
Supply-type	Service Providing	67	1	11	3	82	48.4
	Talent Cultivation	4	12	1	0	17	
	Resource Allocation	6	1	3	4	14	
	Education	7	4	2	7	20	
	Scientific and Technological Information Support	3	2	0	0	5	
Environment-type	Publicity and Education	0	2	18	0	20	41.8
	Broad Participation	5	0	12	0	17	
	Goal Planning	3	5	5	0	13	
	Tactical Approaches	9	1	17	5	32	
	Standardized Supervision	0	16	20	1	37	
Demand-type	Cooperation and Exchange	1	1	0	0	2	9.8
	Market Shaping	3	10	0	2	15	
	government subsidy	0	0	2	1	3	
	Trial Demonstration	4	3	1	0	8	
	Total	112	58	92	23	285	100.0
Sum	Proportion (%)	39.3	20.3	32.3	8.1	100.0	

### 5.1 Policy tool dimension: overall high frequency of use, but varying degrees of preference

#### 5.1.1 Supply-type policy tools: significant factor supply of services but internal structural disproportionality

According to the data in Table 5, it can be seen that the supply-type policy tool has the highest frequency of occurrence, and its percentage is as high as 48.4%. This means that the government is more inclined to use this type of tool and focuses on providing substantial support for the construction related to spiritual older adult care services through the provision of resource elements. The use of the sub-tool on service provision has the highest number of mentions, with a total of 82; in terms of service provision, China currently focuses on mental health services such as psychological counseling and guidance and spiritual comfort in older adult institutions, social legal aid services, and the development of hospice service models, which are policy elements that are conducive to the implementation of the foundations of spiritual older adult care. However, it also faces the problem of unidirectional tilting and unbalanced structural proportions within it. Compared with service provision, the four sub-tools of education and learning, personnel training, resource allocation, and scientific and technological information support are used relatively infrequently: 20, 17, 14, and 5 in that order. The use of these tools is obviously insufficient, making it difficult to effectively meet the urgent need for spiritual older adult care among older groups throughout society. Existing policies are limited in their coverage of universities for the older adult, cultural resources and mental health education; insufficient attention has been paid to the training of professionals in mental health for the older adult; the setting up of preferential policies and the effective provision of cultural and recreational facilities need to be optimized; and scientific and technological information support such as the Internet for services for the older adult needs to be strengthened.

#### 5.1.2 Environment-type policy tools: policy environment is generally good, with mixed levels within groups

Overall, the frequency of the use of environment-type policy tools is on the high side, reaching 41.8%, which creates an open and positive atmosphere for the development of spiritual older adult care, meaning that the government holds a positive attitude toward the development of spiritual older adult care and is committed to shaping a benign development environment for it. This is closely related to the current stage of development in China. Firstly, the government is changing its functions and is committed to creating a limited service-oriented government, so in the development of the older adult care business, it will focus more on the guidance and standardization of older adult care services, and pay more attention to the creation of conditions conducive to the extensive participation of the community. Secondly, spiritual older adult care involves not only medical institutions and nursing institutions, but also social organizations, families and the older adult and other interest groups, and the implementation of related policies requires coordination and linkage among multiple sectors, so at this stage, the Government urgently needs to use environment-type policy tools to break down the barriers to communication among various sectors and subjects. In terms of sub-type policy tools, the government mainly uses standardized regulation and strategic measures, with the content of standardized regulation covering service standards and evaluation mechanisms, the legitimate rights and interests of the older adult, and regulatory control, etc., while the target of support for strategic measures covers the whole society. The diversified application of these two types of tools will help the government to more effectively guide and regulate the work related to spiritual older adult care. In addition, there are 20 tools in the category of publicity and education and 17 tools in the category of extensive participation. In terms of content, they are mainly concerned with actively creating an age-friendly social environment based on respect for and love for the older adult, and encouraging the participation of social forces in spiritual older adult care, but the actual content of the relevant policies is single and the overall number is small, which makes it difficult to provide strong support for the development of spiritual older adult care. The goal-planning tool is the least frequently used, mentioned in only 13 relevant policies; the degree of development of spiritual older adult care is still immature, and the supervisory and guiding role of this type of tool is urgently needed in the exploration process.

#### 5.1.3 Demand-type policy tools: market shaping explored first, demand pull still insufficient

The proportion of demand-type policy tools is 9.8%, which is the least frequently used of the three policy tools. Our government has made good use of market-shaping tools in promoting spiritual older adult care, with a total of 15 articles, introducing policies from optimizing the structure of cultural products, building a healthy cultural tourism industry, and developing age-appropriate intelligent applications, etc., indicating the government’s positive attitude toward supporting the participation of multiple actors in the provision of spiritual older adult care services, and advocates the innovative integration of multi-industry development of services for the older adult that take into account the physical and mental characteristics of the older adult, so as to enrich the intelligent channels through which the older adult can participate in cultural and sports activities, but the enthusiasm for the participation of social capital still needs to be improved. Eight tools of the pilot and demonstration category have appeared, and special pilot policies have been launched one after another for hospice care, cultural older adult care and cultural and tourism older adult care, pointing out the direction of exploration for the development of spiritual older adult care and playing the role of an advanced demonstration. In practice, however, these measures have mainly focused on steadily expanding the standardized hospice care pilot, and lack detailed operational guidance. Three government-subsidized tools appeared, mainly involving resource support, purchase of services, and financial input, etc. Due to the lack of specific management measures such as financial supervision and content evaluation, the corresponding supporting facilities and support systems are not yet perfect, thus making it difficult to ensure that the expected results are achieved. Although cooperation and exchanges are regarded as an important way to learn from experience and innovate for development, there is currently very little content involved, with only two article appearing, and there is an urgent need for further exploration and improvement.

### 5.2 Policy objective dimensions: gaps in policy focus

The percentages of policy objectives in the four categories of spiritual older adult care policies, namely, improving the health service system for the older adult, promoting the high-quality development of the aging cause, building an age-friendly social environment, and meeting the spiritual and cultural needs of the older adult, were 39.3, 20.3, 32.3, and 8.1%, respectively. Among them, improving the older adult health service system involves the most content, indicating the government’s policy preference to optimize service supply and improve the capacity of older adult services. However, since the older adult health service system is still at the stage of favoring the improvement of material services, at the policy level, it mostly advocates the participation of social voluntary organizations, proposes that medical institutions should optimize mental health services, and still lacks the training of professional mental health service personnel. The construction of an age-friendly social environment is also predominant, mainly involving the creation of a social atmosphere of respecting and caring for the older adult, the building of age-friendly communities, and the enhancement of the social participation of the older adult, but there are fewer concrete implementation measures, which still need to be further strengthened and emphasized. As can be seen from Table 5, the policy content on meeting the spiritual older adult care needs of the older adult is the least. Cultural older adult care can improve the comprehensive quality of the older adult group, is an effective way to realize positive aging, and can meet the real needs of the aging society, so efforts should be made to develop cultural older adult care in order to promote the high-quality development of the aging population. Promoting the high-quality development of the aging cause is a requirement for realizing common prosperity in the new era, and high-quality development should be the fundamental direction, with the government further strengthening its policy attention, transitioning to spiritual care on the basis of meeting the basic material life security of the older adult, and realizing the two-way unification of material and spiritual older adult care.

## 6 Analysis of the implementation effect of spiritual older adult care policies based on case studies

In order to more comprehensively and deeply analyze the implementation and effects of the spiritual old-age policy, this study includes qualitative data for additional analysis. By selecting representative regions and organizations as research subjects for case studies, the current implementation status of the relevant policies can be presented from different perspectives, with a view to further exploring the problems and potential influencing factors of the current policies. For example, the Shanghai region has responded positively to the advocacy of spiritual aging policy and vigorously promoted the construction of community cultural activities, creating a number of cultural activity centers for the older adult, and organizing various kinds of cultural performances, calligraphy and painting competitions and other activities on a regular basis. Through in-depth research, it is found that the region has fully integrated community resources and mobilized volunteers in the process of policy implementation, and the spiritual and cultural life of the older adult has been significantly enhanced. However, in the process of organizing the activities, they also faced problems such as lack of funds and insufficient professional talents, which led to a certain impact on the continuity and professionalism of the activities. Meanwhile, in Dalian, a city with a relatively serious degree of aging, the Dalian Ai Yide Nursing Home, as a large-scale nursing home, is different from other simple nursing homes and medical care institutions, in that it has constructed both a nursing home and a care home in the same building, and has actively carried out mental health services with the support of policies, and is equipped with professional psychologists to provide one-on-one psychological guidance services for the older adult. The institution’s mental health services are highly recognized by the older adult, but due to the policy’s uneven distribution of resources, some small nursing homes have difficulty meeting such service standards, highlighting regional and institutional differences in the implementation of the policy.

## 7 Path selection based on policy feedback theory

The development of spiritual older adult care in China is in the primary stage, the number of related policies is extremely limited, and some guidance is also more general, which combines the four kinds of policy feedback effects proposed by Pearson, respectively, from the resource effect, the explanatory effect, the evolutionary effect, and the learning effect to start (31), to explore policy recommendations conducive to promoting the development of spiritual older adult care.

### 7.1 Resource effects: reconciling tensions in the “policy-implementation” relationship

The resource effect refers to the fact that the formulation and implementation of public policy can provide the public with the resources and skills needed to participate in politics, and guide the political perceptions, attitudes and behaviors of the target group, thus influencing the value orientation of new policymaking (32). Currently, policies on spiritual older adult care are limited and focused on positive advocacy, with insufficient training of mental health professionals, limited supply of related service resources and support facilities, and concrete implementation measures that need to be strengthened. As a result, there is a tension between policy and implementation that hinders the implementation of spiritual older adult care policies, and effective measures should be taken to reconcile this complex and evolving tension.

Policymakers should plan their work through the design of policy content and implementation programs, and strengthen the degree of policy implementation in the process of implementation, to ensure that the formulation of public policies is conducive to the participation of citizens, including older persons, i.e., to achieve the visualization and popularization of policies. The first step in policy design is to improve the practicality of the policy, strengthen the relationship with the older adult, and make it gradual and to the point, so as to put forward an implementable policy orientation in accordance with the realities of the older adult, and truly solve the urgent, difficult, worrying and hopeful problems of the older adult; the second step is to allow citizens to have a channel to understand, and to enhance their enthusiasm and participation, so as to make them become the propagandists of the policy in an imperceptible manner. In terms of policy orientation, more content should be involved in enriching the allocation of resources for spiritual older adult care, focusing on expanding the supply of cultural knowledge, mental health and other educational resources in conjunction with the actual needs of older adult groups, and organizing universities for the older adult in a multi-channel, multi-faceted and diversified manner to broaden the universality of education for the older adult; increasing the number of places for leisure and recreational activities for the older adult and the corresponding facilities, and setting up windows for psychological counseling and other medical aids; and design mental health service personnel training programs to make up for the shortcomings of mental health personnel at the grassroots level, and support colleges and universities in opening gerontological psychology and other related programs, establishing related positions and providing appropriately skewed treatment.

### 7.2 Explanatory effects: shaping perceptions of “social-individual” relationships

The explanatory effect refers to the fact that policies, as information in textual form, can shape the perceptions of government officials and citizens through their linguistic expression, content construction and implementation processes, influencing their political attitudes and behaviors, and ultimately affecting the formulation and implementation of future policies. Comprehensively analyzing the content of spiritual older adult care policy as mentioned above, it can be seen that there is a dilemma of insufficient citizens’ awareness of political participation in the current formulation of relevant public policies, which is due to the lack of intercommunication channels between social groups and older adult individuals, i.e., non-older adult groups’ lack of attention and participation in the older adult groups, and at the same time, older adult people’s lack of control over the development trend of the society and their difficulty in adapting to the social situation of the leaping forward, which leads to an information gap between the two groups.

The driving force of public policy on spiritual older adult care should be all citizens, and its core value orientation should be committed to maximizing the realization of the subjective status of older persons. On the one hand, non-older adult groups play a pivotal role as an external force, and in terms of policy propaganda, we should focus on letting all citizens realize the significance of the development of spiritual older adult care for the whole society, i.e., the information expressed in the policy should be easy for the citizens to understand, and we should grasp the key of the matter, remove too many verbal modifications, and stand in the perspective of the masses to explain the meaning of the policy by means of easy-to-understand expressions, and based on the principle of “building and sharing,” the government, social organizations, families and the older adult should form a model of joint participation by multiple subjects; a series of detailed and specific incentives and penalties, as well as supervision and management programs should be introduced in the policy design, so as to fully mobilize public participation and efficiently push forward the implementation and popularization of the public policy on spiritual older adult care. On the other hand, due to individual differences in the needs, willingness and ability of the older adult to spiritual older adult care, there are heterogeneous characteristics, in the policy should be given to the older adult to choose the autonomy of the spiritual older adult care mode, to provide public policy support covering the participation in aging, rights and interests of aging and digital technology for aging and other aspects of the older adult, so that the older adult can be based on their own preferences for aging to choose their own individual welfare to maximize the spiritual older adult care mode (33), and promote the effective integration of citizens of the whole society into the development pace of spiritual older adult care.

### 7.3 Evolutionary effects: conjunction of coupling between “feedback-reinforcement” relationships

The evolutionary effect covers both self-reinforcing and self-defeating effects. The self-reinforcement effect refers to the positive feedback that the policy subject relies on the path of the past policies and formulates new policies by continuously reinforcing the old ones, while the self-weakening effect refers to the negative feedback that the past policies and implementation modes need to be adjusted and updated when the policies have deviations in the process of implementation or are difficult to cope with the new challenges. Overall, the construction of China’s spiritual older adult care service system is at an early stage of development, and although the state has responded to the spiritual needs of the older adult, the construction of content assessment and planning and supervision is not yet sound enough, and urgently needs to be developed and improved.

On the one hand, it is necessary to make use of the positive reinforcement function of the evolution effect, and carry out scientific research and evaluation of the implementation of past policies and their effects during the stage of formulating new policies on spiritual older adult care, and then the policy makers should establish new policy objectives according to the evaluation results and do a good job in the long-term planning for the development of spiritual older adult care, such as drawing up the implementation plan of the policy in the early stage, analyzing the feasibility and cost-effectiveness, and selecting the optimal policy plan and putting it into practice on the basis of the evaluation, including the publicity of the policy, the formulation and implementation of the implementation rules, etc.; in the process of policy implementation, it is necessary to pay attention to the articulation of the policy, and not to be satisfied with half-knowledge, beginning but not the end, and the policy implementers should make the policy take root through real efforts, so as to give better play to the function of positive reinforcement, and to make the policy of spiritual older adult care produce a benign force of inertia. On the other hand, the negative reinforcement function of the policy should be utilized, and the feedback of the policy should be the starting point to pay attention to the reality of the citizens’ feedback. A good system is a system that focuses on people, in the implementation of public policy should focus on mobilizing the political awareness of citizens, and encourage them to actively exercise their right to participate in democratic supervision, effectively preventing arbitrary and blindness due to the subjective factors of the policy implementer, to ensure the rigor of policy implementation and scientific information feedback, to avoid deviations in the implementation of public policy results, in order to play a role in constraining and monitoring. Policies found to be inconsistent with the reality of development should be rectified in a timely manner, so that the formulation-execution-feedback to form a logical closed-loop, so that the cycle continues to promote the long-term development of spiritual older adult care.

### 7.4 Learning effects: accommodating isomorphisms between “space–time-domain” relationships

The learning effect is that policymakers incorporate experiences and lessons learned from past policies into new policy design, and this mechanism can be subdivided into vertical temporal and horizontal spatial policy learning. At present, China’s spiritual older adult care is in the stage of exploratory development, with an imperfect system and a lack of cooperation and exchange with other systems. By utilizing the learning effect of policy feedback theory and learning from domestic and foreign policy experiences in the development of spiritual older adult care from the perspectives of time and space, it will be helpful to form a complete and content-rich Chinese-style spiritual older adult care service system.

From a longitudinal point of view, the family is still the main place of spiritual support for the older adult in China, and the current policy orientation should focus on the family, drawing on the practices of some European countries in the early stages of the process, formulating relevant laws and regulations, and incorporating spiritual support into the same system of evaluation as the career prospects and social credibility of the children, so as to give filial piety a new connotation and the spirit of the times. In terms of the proportion of the older adult population, Singapore and China are both countries with a high degree of aging in Asia, and its early advocacy of the happy aging model for the senior citizens and the introduction of relevant institutional safeguards are also worthwhile for China to learn from, such as through the provision of employment training, extension of retirement and other policies to encourage the older adult to maintain interaction with the community. It is necessary to learn from the development concepts of these countries based on China’s national conditions, and learn from their lessons, so as to minimize detours in macro policy control and accelerate the development process of spiritual older adult care in China. From a horizontal spatial perspective, we can actively cooperate and exchange with the domestic spiritual older adult care services in the development of good advanced regions, the use of data thinking mode to build a visual communication platform, open and tolerant mentality to carry out exchanges in the industry, the relevant policies introduced by these advanced regions to objectively analyze, and on this basis, combined with the local conditions of each region to develop the characteristics of the region’s spiritual older adult care, never copy the phenomenon of formalism, and build a spiritual older adult care service system with Chinese characteristics based on the concepts of China’s excellent traditional culture.

## 8 Discussion

The results of the study show that in the dimension of spiritual old-age policy tools, there is an imbalance in the internal structure of the supply-type tools, and although their service provision is relatively emphasized, they are weak in the areas of education and learning and personnel training, making it difficult to meet the diversified needs of the older adult. Uneven development within the group of environment-based tools, and insufficient tools in the categories of publicity, education and broad participation, leading to low social awareness of and participation in spiritual aging and limiting the implementation of policies. There is a shortage of demand-based tools, and market vitality has not been fully stimulated, hindering the diversified development of the industry. In the dimension of policy objectives, insufficient attention has been paid to the spiritual and cultural needs of older persons, which is not conducive to the realization of positive aging and affects the quality of life of older persons and social harmony.

It cannot be ignored that this study has certain limitations. In the selection of the research sample, it mainly focuses on policy texts at the national level, with less coverage of local policies. China has a vast territory, and there are significant differences between different regions in terms of the degree of aging, the level of economic development, and the cultural background, etc. Local policies play a key role in the practice of spiritual aging. The failure to fully incorporate local policies makes it possible that the results of the study may not fully reflect the actual situation of spiritual aging policies in China, and the generalizability and applicability of the study’s conclusions are affected. Policies are affected by a variety of factors in the process of implementation, and there may be discrepancies between actual effects and policy expectations. Although existing studies were referred to in the construction of the two-dimensional analytical framework, the categorization of policy tools and objectives may not cover all aspects of spiritual old-age policy, and there is a certain degree of subjectivity.

Nevertheless, this study has important practical implications. In terms of policy formulation, it clearly points out the shortcomings of policy tools and target dimensions, providing valuable references for policymakers and helping them to adjust and improve policies in a targeted manner. In terms of promoting the development of spiritual older adult services, the issues revealed in the study can guide the community to pay attention to the shortcomings of spiritual older adult services, promote the rational allocation of resources, attract the participation of more social capital, promote the construction of the spiritual older adult service system, and improve the accessibility and quality of services. In the field of academic research, this study enriches the content and methodology of spiritual older adult policy research, provides ideas and references for subsequent research, and helps attract more scholars to pay attention to this field and promote the in-depth development of academic research.

Existing literature mostly focuses on one aspect of spiritual old-age care, but this study takes policy tools and objectives into consideration to construct a two-dimensional analytical framework for spiritual old-age care policy research and fill in the gaps in related research. Through comprehensive and in-depth analysis of the policy content, it reveals the current situation and potential problems of China’s spiritual old-age policy, and provides empirical data support for domestic and foreign scholars to understand China’s spiritual old-age policy, with a view to facilitating academic exchanges and the sharing of research results. Meanwhile, the application of policy feedback theory enhances the practicability of the policy analysis results and provides new perspectives and methods for the subsequent research on spiritual older adult policy.

## 9 Conclusion

This study constructs a two-dimensional analysis framework of policy tools-policy objectives, and after analyzing 71 spiritual older adult policy texts at the national level from 2013 to 2024, it is found that: there is an imbalance in the internal structure of supply-type tools, uneven development within the group of environment-type tools, and a shortage of demand-type tools in the policy tools dimension; in the policy objectives dimension, the Improving the older adult health service system and building an age-friendly social environment receive more attention, but there are fewer policy contents related to meeting the spiritual and cultural needs of the older adult. Based on this, from the resource, explanation, evolution and learning effects of policy feedback theory, the following more targeted and practical policy recommendations are put forward: first, optimize supply-based policy tools. In terms of service provision, the current focus on mental health services in nursing facilities has effectively met the basic mental needs of some older people, but its internal structure should be adjusted in order to better fulfill its role. Increase investment in education and learning tools, such as the development of specialized mental health teaching materials for the older adult, and the development of diversified courses through communities, senior universities and other platforms to enhance the spiritual and cultural literacy of the older adult. Strengthening talent training, encouraging colleges and universities to cooperate with older adult care organizations to set up internship bases, and cultivating talents with both knowledge of geriatric psychology and professional service skills. Reasonable allocation of resources, accurate layout of cultural and recreational facilities according to the degree of aging and demand characteristics of different regions, while strengthening scientific and technological information support, using intelligent equipment to provide convenient spiritual and cultural services for the older adult, such as online psychological counseling and live broadcasting of cultural activities. Second, strengthening environment-oriented policy tools. The tools of normative regulation and strategic measures have worked to a certain extent, but the level of tools of publicity and education and broad participation needs to be further improved. In terms of publicity and education, new media platforms can be utilized to produce lively and interesting short videos and cartoons on popular science of mental aging in order to increase social attention. At the same time, social forces should be encouraged to participate widely by organizing thematic activities on mental aging, such as the “Month of Caring for the Older adult’s Mental Health.” Improvement of goal-planning tools, setting clear milestones and assessment indicators for the development of mental health care, guiding the direction of policy implementation, and ensuring the orderly advancement of mental health care. Third, activate demand-based policy tools. The government should increase subsidies, set up a special fund for mental older adult care, and clarify the standard and scope of subsidies, such as financial incentives for organizations providing quality mental older adult care services and tax incentives for enterprises participating in mental older adult care services, etc., as well as strengthening financial supervision and effect evaluation. Expanding the scope of pilot demonstrations, in addition to hospice care, conducting more pilot projects in areas such as cultural older adult care and technological assistance for the older adult, and summarizing and promoting experiences in a timely manner. Strengthening cooperation and exchanges, building a platform for multi-party cooperation among older adult care institutions, enterprises and social organizations, promoting resource sharing and experience exchange, and attracting more social capital to invest in the field of spiritual older adult care. Fourth, promote the synergistic effect of policy tools. Different types of policy tools should cooperate and synergize with each other. Supply-type policy tools to provide basic resources and services for demand-type policy tools to attract social capital to create conditions; environmental policy tools to create a favorable environment to ensure the effective implementation of supply-type and demand-type policy tools. Establish a coordinating mechanism for policy tools, led by relevant government departments, to regularly organize joint meetings involving all parties to discuss implementation plans and solutions to problems in mental old-age policies. In formulating policies, full consideration should be given to the complementary nature of different tools, so as to avoid policy conflicts and wasted resources, and to form a strong synergy in promoting the development of mental old-age care.

The two-dimensional analytical framework constructed in this study provides a new perspective for the study of spiritual old-age policy, revealing the current situation and problems of the policy through quantitative analysis, and providing a reference for academic research and policy formulation. However, the study has certain limitations, such as the sample does not fully cover local policies, the analytical framework is subjective, etc. The policy recommendations based on the policy feedback theory have not been empirically studied to assess their actual effects, and future research will be conducted in the following directions: firstly, we will expand the sample scope to include more local policies, so as to study in depth the differences and commonalities of spiritual old-age policies in different regions; secondly, we will incorporate empirical research, actively prepare resources, establish cooperative relationships with relevant organizations, and collect richer data for evaluation through long-term tracking of the policy implementation process, so as to further validate and improve the policy recommendations, and to provide more practically guiding results for the development of spiritual old-age policies; thirdly, we will further optimize the analysis framework to more comprehensively and accurately analyze the spiritual old-age policy, so as to provide more powerful support for improving the spiritual old-age policy system in China and enhancing the quality of spiritual life of the older adult.

## Data Availability

The original contributions presented in the study are included in the article/supplementary material, further inquiries can be directed to the corresponding author.
